# Ordered weighted evaluation method of lifting operation safety risks considering coupling effect

**DOI:** 10.1038/s41598-024-56039-9

**Published:** 2024-03-09

**Authors:** Kesheng Yan, Lianghai Jin, Xiaoyun Yu

**Affiliations:** 1 College of Hydraulic & Environmental Engineering, China Three Gorges University, Yichang, 443002 Hubei China; 2 Safety Production Standardization Evaluation Center of China Three Gorges University, Yichang, 443002 Hubei China

**Keywords:** Lifting operation, Safety risk, Coupling effect, Interaction matrix, Joint ordered weighted operator, Civil engineering, Energy infrastructure

## Abstract

In order to accurately evaluate the safety risk degree of lifting operations and improve the safety control level of lifting operations, firstly, Work Breakdown Structure (WBS) is used to decompose the construction process of lifting operations, Risk Breakdown Structure (RBS) is used to decompose the safety risk, 16 safety risk factors of lifting operations are identified, and the risk evaluation system is constructed. Then, the safety risk assessment model of lifting operations is constructed by integrating risk coupling theory, joint ordered weighting operators, and an interaction matrix to identify key risk factors and safety risk levels. Finally, taking the lifting operation construction project of Yangfanggou Hydropower Station as an example, the evaluation index system and method are applied. The results show that the high-risk safety risks of the lifting operation construction of the project are: 'Low safety awareness causes workers to operate illegally *X*_23_', 'Operation error caused by illegal command *X*_33_', 'Workers' risky work caused by low safety awareness *X*_34_', 'Edge, high and climbing operation protection is not in place *X*_36_', and 'Improper binding of components caused by workers' illegal operation *X*_25_'. The construction of the lifting operation poses a significant risk level, and the evaluation results are consistent with the actual situation. The rationality of the evaluation system and model constructed in this paper can provide a reference for the safety management and control of the construction site of the lifting operation and the safety of the lifting operation.

## Introduction

Lifting operation is a kind of labor combination which takes the lifting driver as the main body, cooperates with the cable workers and signal workers, and aims to complete the vertical transportation task. It is an indispensable and important means for the construction of large-scale energy, major chemical industry, high-rise buildings and other projects^1^. As one of the most important pieces of special equipment, a crane is one of the most important means of vertical lifting and horizontally moving materials and is widely used in infrastructure construction and other fields^2^. In the process of lifting operations, due to the complex man–machine interface, high task difficulty, and uncertain information, there is a potential high risk^3^, which makes the causes of lifting injury accidents such as overload, collision, and operation error highly complex, which can easily lead to lifting injury accidents, resulting in serious consequences such as casualties and property losses^4^. The safety evaluation index system of lifting operation plays an important role in the safety control of lifting operation. Through the safety evaluation of each link of the lifting operation, the system identifies the possible safety hazards, so as to take corresponding measures to prevent the occurrence of accidents in advance. Therefore, it is of great significance to construct a reasonable and scientific lifting operation risk index system and method to evaluate and study the lifting operation risk for the safety risk management of the whole process of lifting operation construction.

Lifting operations are complex systems composed of humans, machines, the environment, management, and other factors^5^. Different factors play different roles in the cause of the accident. Human factors mainly include human unsafe behavior^6^, which is the main reason for the occurrence; the problem of management factors is the premise of the unsafe behavior of people and the unsafe state of things. The coupling effect of various accident causes leads to the occurrence of lifting accidents^7^. The safety state of lifting operations changes with the change of human factors, material factors, environmental factors, and management factors. Previous studies mainly focus on the identification of risk factors in lifting operations, and there are few studies on the coupling effect among risk factors and safety risk assessment. The main risk factors identification methods include expert interviews, questionnaires, case studies, technical checklist reviews, etc. The research on the relationship among risk factors mainly adopts Bayesian network reasoning and complex network theory. For exanple, Zhang^8^ combined system thinking and complex networks for the first time, divided tower crane accidents into 6 levels, identified 34 disaster-causing factors and 10 accident types, and identified 7 key factors and 3 critical paths of tower crane accidents in China. Zhang^9^ identified the key causes of tower crane accidents through systematic thinking and case analysis, including workers wrong behavior, insufficient safety training, insufficient safety inspection, low safety awareness, and poor management of safety engineers. Ding Ke et al.^10^ analyzed the tower crane injury accident based on the accident cause theory, summarized the accident causes of the tower crane injury accident, and found that the project's own conditions and human effects were the main causes of the tower crane accident. Zheng Xiazhong et al.^11^ studied the causes of accidents affecting lifting operations through the Delphi method and concluded that more attention should be paid to those factors that are assessed as highly affecting on-site safety due to tower crane work. Most scholars use complex network parameters^12,13^, Bayesian networks^14^, frequency, etc., to evaluate the causes of lifting accidents, and less consideration is given to the coupling relationship among accident causes. The coupling relationship among accident causes has a huge impact on the occurrence, development, and severity of lifting accidents. In the past, the research on the interaction among risk factors is easy to receive the influence of subjective judgment. The continuous-ordered weighted averaging (C-OWA) operator of combination number realizes the scientific weighting of the subjective data of the survey by improving the data set of the ordered weighted averaging operator. In addition, previous studies have paid less attention to the construction process, and most of them identify the cause of the accident based on the lifting operation accident report. For example, Yang and Jin^15^ identified the causes of lifting accidents from a large number of accident reports, and identified the causes of key accidents according to the topological potential theory. Based on a large number of lifting accident reports, Wu et al.^16^ used text mining technology to identify risk factors and simulate their coupling relationship. Due to the high technical requirements of lifting operations, the complex construction environment, and the large number of personnel and machinery on the construction site, the lifting operation process is full of uncertainty. However, the previous research has laid a solid foundation for the safety control of lifting operation, but paid a insufficient attention to the construction process of lifting operation and the evaluation of the influence among the risk factors of lifting operation is subjective.

In view of this, according to accident cause theory and system theory, this paper uses work breakdown structure (WBS)^17^ to decompose the lifting operation process, risk breakdown structure (RBS)^18^ to decompose the safety risk of lifting operation, and constructs the safety risk evaluation system of lifting operation with 16 secondary indexes. Considering the coupling effect among the causes of lifting injury accidents, the continuous-ordered weighted averaging (C-OWA)^19^ operator and the interaction matrix theory^20^ are introduced to construct a risk coupling evaluation model for the causes of lifting injury accidents. The C-OWA operator is used to weaken the influence of subjective factors on experts to obtain more objective evaluation results. At the same time, the interaction among accident-causing factors is considered to identify key safety risk factors and propose prevention and control strategies. Then, the risk level of the lifting operation of the project is evaluated to guide the safety control of the lifting operation site and provide a scientific and reasonable assessment method for the risk assessment of the lifting operation. The safety evaluation method of lifting operation based on C-OWA proposed in this paper can provide more comprehensive, accurate and objective evaluation results for the knowledge system, and can provide more scientific and reasonable decision-making basis for decision makers.

## Establishment of construction safety risk assessment index system

In order to comprehensively identify the safety risks of lifting operations, a systematic risk assessment index system is constructed. Through literature research and field investigation, the two-level risk identification based on WBS-RBS is carried out under the guidance of Work Breakdown Structure (WBS)^21^ and Risk Breakdown Structure (RBS)^22^. The safety risk of lifting operations is identified by four steps: work breakdown structure, risk breakdown structure, coupling interaction analysis, and index correction.

1. Work breakdown structure: According to the principle of WBS work decomposition, the construction process of lifting operations is decomposed into two levels^23^: the first level WBS is decomposed into a lifting preparation stage, a component binding stage, a component lifting stage, and a component unloading stage according to the construction process; combined with the working characteristics of each stage of lifting operations, each level 1 WBS is decomposed into a level 2 WBS according to the characteristics of the process^24^. The decomposition results of level 2 WBS are shown in Table 1.

**Table 1:** Two-level WBS decomposition of lifting operation process.

Lifting preparation: *W*_1_	Component binding: *W*_2_	Component lifting: *W*_3_	Component unloading: *W*_4_
Lifting equipment selection and layout: *W*_11_	Conceptual design: *W*_21_	Lifting plan formulation: *W*_31_	Unloading scheme formulation: *W*_41_
Construction site layout: *W*_12_	Selection of hoisting tools: *W*_22_	On-site command lifting: *W*_32_	On-site unloading of components: *W*_42_
Component transport: *W*_13_	On-site binding of components: *W*_23_	On-site lifting of components: *W*_33_	–
Component unloading: *W*_14_	–	–	–
2. Risk decomposition structure: According to the principle of RBS risk decomposition^25^, the risk source of lifting operation construction is decomposed into two levels of risk, and the first level of RBS is decomposed into 'man–machine environment management' according to the characteristics of the construction project. Combined with the first-level decomposition characteristics, all potential risk factors are determined to construct a second-level RBS, and the second-level RBS decomposition results are shown in Table 2.

**Table 2:** Two-level RBS decomposition of lifting operation process.

Man risk: *R*_1_	Machine risk: *R*_2_	Material risk: *R*_3_	Method risk: *R*_4_	Environment risk: *R*_5_
Low safety awareness: *R*_11_	Equipment out of the factory with disease: *R*_21_	Poor quality of the suspension: *R*_31_	Improper investigation of safety hazards: *R*_41_	Extreme weather conditions: *R*_31_
Wildcat operatio: *R*_12_	Balance of plant fault: *R*_22_	Poor quality of spreader: *R*_32_	Improper safety education and training: *R*_42_	Cross operation: *R*_32_
Unlicensed employment: *R*_13_	Improper maintenance: *R*_23_	–	There is no full-time supervisor on site: *R*_43_	Poor hydrogeological conditions: *R*_33_
Improper use of safety protective equipment: *R*_14_	Limiter failure: *R*_24_	–	Improper special construction scheme: *R*_44_	Lack of on-site warning signs: *R*_34_
Violate commanding: *R*_15_	Improper equipment layout: *R*_25_	–	Improper disclosure of construction technology: *R*_45_	–
Physical discomfort on duty: *R*_16_	–	–	–	–
Risk Operation: *R*_17_	–	–	–	–
3. Coupling interaction analysis^26^: The WBS work decomposition in Table 1 is set as a column, and the RBS risk decomposition in Table 2 is listed as a row, and the risk coupling interaction matrix of lifting operation construction is established. If the risk exists, it corresponds to the horizontal and vertical coupling interaction mark '1', otherwise it marks '0'. Taking the formwork preparation (*W*_1_) as an example, *W*_11_ interacts with *R*_24_ and *R*_43_ to generate the risk of 'improper safety supervision causing unreasonable equipment selection and layout', and so on. Traversing the whole 'risk decomposition coupling interaction matrix', the two-level decomposition coupling analysis of WBS-RBS is obtained as shown in Table 3. Ten experienced experts with more than 5 years of experience in relevant construction, management and teaching were invited to check and correct the results (5 project managers, 3 lifting construction personnel, 2 construction safety management research professors). Combined with the results of expert correction, the coupling interaction matrix of WBS-RBS for lifting construction was finally obtained.

**Table 3:** Two-level WBS-RBS decomposition of lifting operation process.

	*W*_11_	*W*_12_	*W*_13_	*W*_14_		*W*_11_	*W*_12_	*W*_13_	*W*_14_		*W*_11_	*W*_12_	*W*_13_	*W*_14_
*R*_11_	0	0	1	1	*R*_22_	0	0	0	0	*R*_43_	0	0	1	0
*R*_12_	0	0	1	1	*R*_23_	0	0	0	0	*R*_44_	0	1	1	0
*R*_13_	0	0	1	1	*R*_24_	0	0	0	0	*R*_45_	0	0	0	0
*R*_14_	0	0	0	0	*R*_25_	0	1	0	0	*R*_51_	0	0	0	0
*R*_15_	0	0	0	0	*R*_31_	0	0	1	0	*R*_52_	0	1	0	0
*R*_16_	0	0	1	1	*R*_32_	0	0	1	0	*R*_53_	0	0	0	0
*R*_17_	0	0	1	1	*R*_41_	0	1	0	0	*R*_54_	0	0	0	0
*R*_21_	0	0	0	0	*R*_42_	0	1	0	0	–	–	–	–	–
4. Index correction: Based on the initial evaluation index system constructed by WBS-RBS^27^, combined with the opinions and suggestions of experts in the field of lifting operation construction and the experience of relevant practitioners, the initial index system is judged, supplemented, and revised, and the safety risk assessment index system of lifting operations is systematically summarized. The complete index system is shown in Table 4.

**Table 4:** Climbing formwork construction safety risk evaluation index system.

Phase	Index	Indicator description	Phase	Index	Indicator description
Lifting preparation	*X*_11_	The unqualified comprehensive acceptance system leads to the equipment and components leaving the factory with diseases	Component lifting	*X*_31_	Equipment safety device failure leads to overload lifting
	*X*_12_	Unreasonable equipment layout causes potential safety hazard		*X*_32_	Defects in the equipment lead to operational errors
	*X*_13_	Unreasonable storage of equipment and components causes quality problems		*X*_33_	Operation error caused by illegal command
Component binding	*X*_21_	Improper binding of components caused by workers ' illegal operation		*X*_34_	Workers ' risky work caused by low safety awareness
	*X*_22_	Improper safety supervision leads to quality defects in slings		*X*_35_	The lifting is not carried out according to the scheme, resulting in component collision
	*X*_23_	Low safety awareness causes workers to operate illegally		*X*_36_	Edge, high and climbing operation protection is not in place
	*X*_24_	Failure to implement the scheme leads to improper binding of components		*X*_37_	Improper binding causes the component to fall off
	*X*_25_	Technical disclosure leads to improper binding of components	Component unloading	*X*_41_	Unreasonable unloading of components caused by improper construction scheme and illegal operation of workers

## Lifting operation safety risk assessment model

### Model establishment

**Figure 1:**
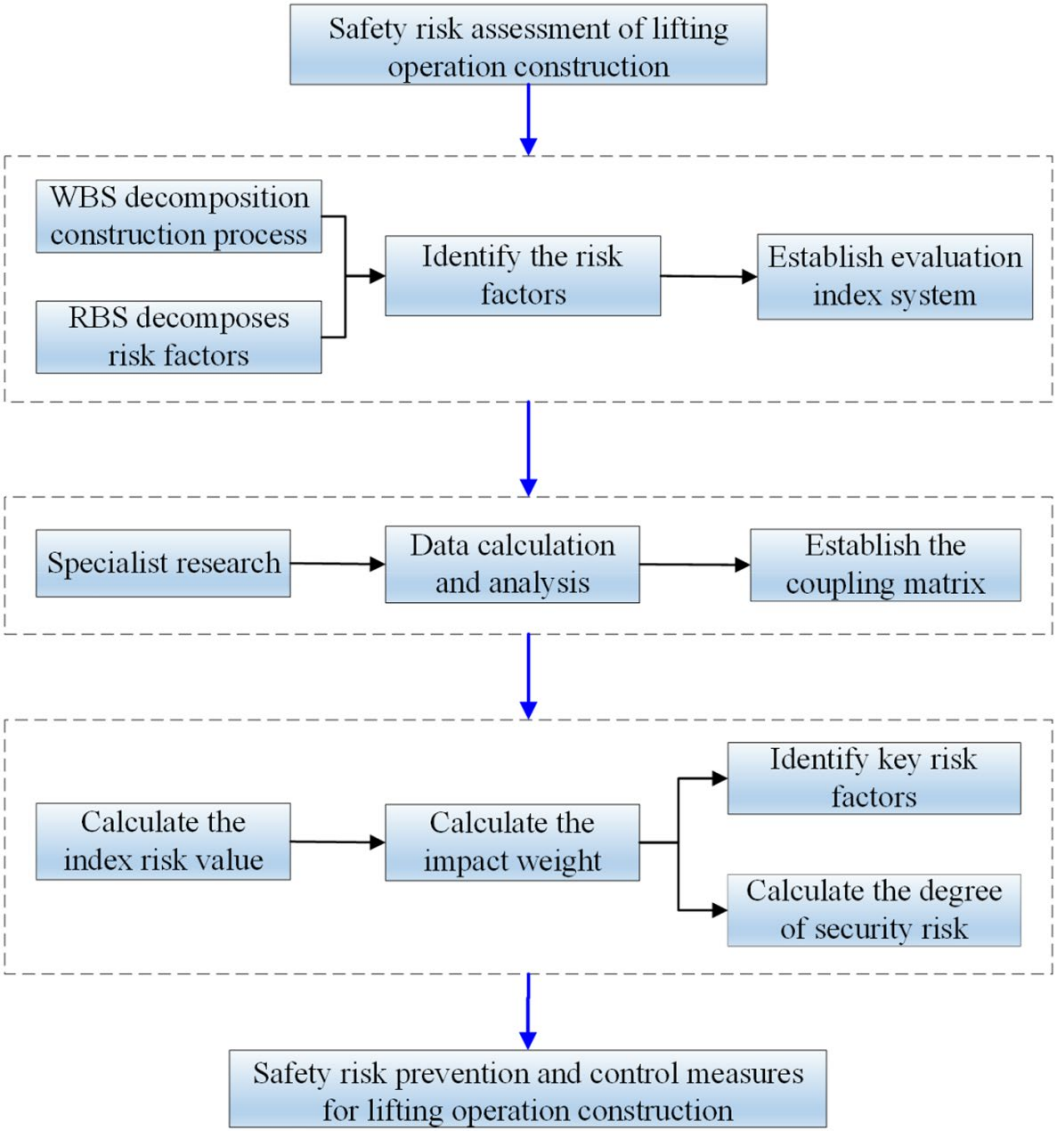
Risk assessment model technology roadmap.

The process of safety risk assessment for lifting operations is as follows: Firstly, WBS and RBS are combined to decompose the construction process and safety risk of lifting operations. According to the experience of lifting operation construction experts, the safety risk factors are identified, and the evaluation index system for lifting operation construction is established. Then, according to the expert survey and using the C-OWA operator to calculate the survey data, the coupling matrix of the risk factors of the lifting operation construction is constructed, and the weight of the risk factors is calculated to excavate the key risk factors of the lifting operation construction^28^. Finally, the risk evaluation coefficient of lifting operation construction is calculated, and the risk level is determined. The technical route of the evaluation model is shown in Fig. 1.

### Orderly weighted aggregation of lifting operation safety risk

The combination ordered weighted averaging (C-OWA) operator^29^ combines the weight and sample data randomly, which effectively reduces the subjective experience value of experts evaluation of lifting operation safety risk factors shown in Table 2 and makes the research results more objective and scientific. In order to obtain more objective survey results, the C-OWA operator is used to calculate the expert score. The calculation steps for the elements on the main diagonal are as follows:

Invite m experts to score the indicators (using a 5-point system, the greater the score, the greater the risk)^30^, and set m experts to score to obtain the initial evaluation matrix *A* = [*a*_1_, *a*_2_, …, *a*_m_].

The initial evaluation matrix A is sorted in descending order and numbered from 0 to obtain a descending-order matrix, *B* = [*b*_0_, *b*_1_, *b*_2_, …, *a*_m−1_] and *b*_0_ ≥ *b*_1_ ≥ *b*_2_ ≥ ⋯ ≥ *b*_m−1_. Let the weighted weight vector of bh in matrix *B* be *φ* = [*φ*_1_, *φ*_2_, …, *φ*_m_], as shown in Eq. (1):

1 $$\phi_{h + 1} = \frac{{C_{{{\text{m}} - 1}}^{h} }}{{\sum\nolimits_{h = 0}^{m - 1} {C_{{{\text{m}} - 1}}^{h} } }} = \frac{{C_{{{\text{m}} - 1}}^{h} }}{{2^{m - 1} }}$$

where, *φ*_h+1_ is the weighted weight of the descending sequence *b*_h_; *C*^h^_*m*−1_ is the number of permutations, *h* = 0, 1, 2, …, *m*−1.

The comprehensive weight set of evaluation index *Q* = [*Q*_1_, *Q*_2_, …, *Q*_i_] is obtained by using *φ*_h+1_ and *b*_h_ weighted calculation, as shown in Eq. (2):

2 $$\begin{aligned} Q_{i} & = \phi_{1} \cdot b_{0} + \phi_{2} \cdot b_{1} + \cdots + \phi_{m} \cdot b_{m - 1} \\ & = \sum\limits_{h = 0}^{m - 1} {[(\phi_{h + 1} )^{T} \cdot b_{h} ]} \\ \end{aligned}$$

where, *Q*_i_ is the comprehensive weight of the* i*-th index, *h* = 0, 1, 2, …, *m*−1.

Calculate the relative weight of evaluation index $\phi_{i}$:

3 $$\phi_{i} = \frac{{Q_{i} }}{{\sum\nolimits_{i = 1}^{m} {Q_{i} } }}$$

### The mutual coupling effect of lifting operation safety risk

The interaction matrix^31^ is used to identify the key risks of lifting operations. The interaction matrix is a factor interaction matrix composed of many factors in a certain way, which aims to solve the complex problem of multiple influencing factors in the system^32^. Taking the 16 risk indicators of lifting operation construction as an example, the 16 influencing factors are listed on the main diagonal line. The strength of the interaction between the two factors is encoded by 1–5, which represents the interaction of no (1), weak (2), medium (3), strong (4) and strong (5), and is listed on the non-diagonal line to form a 16*16 interaction matrix^33^. The interaction matrix is shown in Table 5.

**Table 5:** Crane injury accident causation interaction matrix.

*X*_1_	*X*_12_	*X*_13_	…	Column *i* : The influence of other main factors on *X*_j_ on the main diagonal	
*X*_21_	*X*_2_	*X*_23_	…		
*X*_31_	*X*_32_	*X*_3_	…		
…	…	…	…	…	…
Row *i* : The influence of *X*_i_ on other main factors on the main diagonal			…	*X*_15_	*X*_15, 16_
			…	*X*_16, 15_	*X*_16_

According to the Table 5, in the matrix of 16*16, *X*_1_, *X*_2_, …, *X*_16_ is the first lifting operation safety risk index; *X*_*ij*_ represents the degree of influence of *X*_*i*_ on the system generated by *X*_*j*_. Based on the interaction matrix, the influence of the i-th factor on other factors, the total influence degree, and the weight set can be further calculated, which are *C*_*i*_, *E*_*i*_ and *K*_*i*_, respectively, as shown in formulas (4)–(6):

4 $$C_{i} = \sum\limits_{j = 1}^{16} {X_{ij} (j = 1,2, \ldots ,16)}$$

5 $$E_{i} = \sum\limits_{j = 1}^{16} {X_{ji} (j = 1,2, \ldots ,16)}$$

6 $$K_{i} = \frac{{C_{i} + E_{i} }}{{2\sum\nolimits_{j = 1}^{n} {X_{ij} } }}(j = 1,2, \ldots ,16)$$

Through Eqs. (3–5), after calculating the influence weight set *K* of the safety risk of lifting operation, combined with the comprehensive weight set *Q* of each evaluation index risk in Eq. (2), the safety risk degree *P* of lifting operation can be calculated from Eq. (6).

7 $$P_{i} = \sum\limits_{i = 1}^{k} {k_{i} \times Q_{i} }$$

Through the calculation of Eqs. (4)–(7), the weight of lifting operation safety risk factors can be quantified and ranked, the key risks can be identified, and the basis for decision-making for risk control can be provided.

### Safety risk level assessment of lifting operation

In order to accurately classify the safety risk of lifting operations and implement refined safety early warning, the risk assessment system for lifting injury accidents is constructed according to accident cause theory and WBS-RBS decomposition. The risk coupling theory^34^, C-OWA operator theory, and interaction matrix theory are introduced to construct the safety risk assessment model of lifting operations and quantitatively calculate the risk degree of lifting operation construction* P*^35^. Referring to the relevant standards and research literature, the risk degree P value is divided into I, II, III, IV, and V levels^36^, and the safety risk level of lifting operations is characterized as shown in Table 6.

**Table 6:** Safety risk level of crane operation.

*P* value	Risk degree	Risk level
[0.80, 1.00]	Extremely dangerous	I
[0.60, 0.80)	Highly dangerous	II
[0.40, 0.60)	Significant dangerous	III
[0.20, 0.40)	General dangerous	IV
[0.00, 0.20)	Very weak dangerous	V

## Engineering example analysis

### Introduction of engineering

The Yangfanggou Hydropower Station is located on the middle reaches of the Yalong River in Muli County, Liangshan Yi Autonomous Prefecture, Sichuan Province (part of the project area is located in Jiulong County, Ganzi Prefecture). The dam site of the power station is about 450 m away from the downstream Yangfanggou estuary. The Yangfanggou Hydropower Station is the sixth stage of the seventh-level development of the first reservoir in the middle reaches of the Yalong River. It is 37 km from the Mengdigou Hydropower Station and 33 km from the Kala Hydropower Station. The dam site of the power station is about 235 km away from the highway in Xichang and about 156 km away from Muli County.

Yangfanggou Dam is a concrete double-curvature arch dam with a maximum dam height of 155.0 m. The total concrete of Yangfanggou Dam is about 977 thousand m^3^, and the peak strength of concrete pouring is about 54 thousand m^3^ per month. According to the results of the bidding design, combined with the strength of concrete pouring, and considering the lifting requirements of the metal structure and leaving a certain margin, three 30 t translational cable cranes are selected to be responsible for the concrete pouring of the dam of the project.

The plane layout pattern of the Yangfanggou cable crane is as follows: the main tower platform and feeding platform of the cable crane are arranged on the left bank, and the auxiliary tower platform is arranged on the right bank; the cable crane platform on the left bank is arranged at an elevation of 2190 m, and the cable crane platform on the right bank is arranged at an elevation of 2185 m. The cable crane span is about 416 m, and the excavation width of the cable crane platform on the left and right banks is 20 m and 15.5 m, respectively. The feeding platform is arranged at an elevation of 2102 m at the top of the dam, and the total width of the platform is 20–50 m.

### Risk assessment of lifting operation of Yangfanggou hydropower station project

#### Risk analysis of the lifting operation of Yangfanggou hydropower project

Yangfanggou is located in the western Sichuan Plateau climate zone. Affected by the southwest monsoon, the temperature difference between day and night is large. April–October is the rainy season, and the rainfall accounts for more than 97% of the year. In summer, the rainfall is concentrated and the frequency of rainstorms is high, which makes it easy to cause geological disasters and affect the lifting operation. The project is located in the middle reaches of the Yalong River, and the complex geological conditions also affect the lifting operation. According to the field investigation and expert interview of the project, combined with the analysis of the risk evaluation index system of the cause of the lifting injury accident, the project is located on a plateau, the environment is harsh, the geological situation is complex, the field construction personnel are numerous, and the field construction machinery is numerous. There is not only a single risk impact on the construction site but also a coupling effect of various accident causes, which has a large hidden danger. Therefore, based on the lifting operation risk evaluation index system and evaluation model, the safety risk of the lifting operation of the project is coupled and evaluated, which provides the basis for on-site safety management and control.

#### Cable engineering risk value calculation based on the C-OWA operator

According to the constructed risk assessment index system for lifting injury accidents, combined with the operation of the cable crane in the project, 15 experts with more than 10 years of experience in the construction site of lifting operations were invited to score the risk of lifting operation accidents to obtain the initial evaluation matrix A. The score is between 1 and 5. The higher the score, the greater the risk of the index, which includes five project managers, six operators, and two signal workers. In addition, two experts are university teachers who have been engaged in teaching and scientific research in construction safety management for more than 15 years. A total of two rounds of questionnaire distribution and recovery activities were conducted, with a 100% recovery rate. The positive coefficients of the invited experts were qualified. The reliability and validity of the results of lifting operation accidents were tested by the reliability coefficient method and the factor analysis method. The Cronbach's alpha coefficient was 0.993 > 0.800, the validity coefficient KMO was 0.835 > 0.800, and the reliability and validity test results were good.

For example, the scores of the 15 experts in the indicator *X*_23_ are *a* = [3, 4, 4, 4, 4, 4, 4, 4, 4, 5, 5, 5, 5, 5, 5]. The ranking expert score vector *b* is obtained by ranking a in descending order, then *b* = [3, 4, 4, 4, 4, 4, 4, 5, 5, 5, 5, 5]. The weight *φ* corresponding to *b* is obtained from Eq. (1), that is, *φ* = [1/2^12^, 14/2^12^, 91/2^12^, 364/2^12^, 1001/2^12^, 2002/2^12^, 3003/2^12^, 3432/2^12^, 3003/2^12^, 2002/2^12^, 1001/2^12^, 364/2^12^, 91/2^12^, 14/2^12^, 1/2^12^]. Moreover, the risk value of *X*_23_ is obtained from (2): *Q* = 4.2119. Similarly, the risk values of the remaining 13 risk indicators can be obtained. The results are shown in Table 7.

**Table 7:** Weight and importance of risk factors in lifting operation construction.

Index	Risk degree	C	E	C + E	Weight	Weight sorting	Importance degree	Importance sorting
*X*_11_	2.7823	0.00	14.02	14.02	0.0115	10	0.0319	9
*X*_12_	2.3225	6.0000	7.08	13.08	0.0106	13	0.0247	12
*X*_13_	1.3297	0.00	7.32	7.32	0.0057	15	0.0076	15
*X*_21_	2.9778	21.32	7.81	29.13	0.0229	3	0.0683	5
*X*_22_	2.6601	0.00	22.17	22.17	0.0180	5	0.0479	7
*X*_23_	4.1833	52.11	4.05	56.16	0.0459	1	0.1919	1
*X*_24_	2.4722	8.16	6.25	14.41	0.0115	11	0.0283	10
*X*_25_	2.4307	8.09	5.73	13.82	0.0101	14	0.0279	11
*X*_31_	2.5263	11.27	5.28	16.55	0.0131	8	0.0331	8
*X*_32_	4.9935	8.07	7.24	15.31	0.0123	9	0.0613	6
*X*_33_	4.2119	53.27	1.35	54.62	0.0442	2	0.1863	2
*X*_34_	4.9102	12.53	7.20	19.73	0.0156	7	0.0764	3
*X*_35_	1.6755	9.14	11.04	20.18	0.0164	6	0.0274	12
*X*_36_	3.4713	19.25	7.31	26.56	0.0213	4	0.0739	4
*X*_37_	4.8511	13.26	6.90	20.16	0.0114	12	0.0261	13
*X*_41_	2.8623	0.00	7.22	7.22	0.0057	16	0.0164	14

It can be seen from Table 7 that the importance degree takes into account the degree of influence and the risk value, which can effectively identify the key security risks. The top five risk importance rankings are: 'Low safety awareness causes workers to operate illegally *X*_23_', 'Operation error caused by illegal command *X*_33_', 'Workers' risky work caused by low safety awareness *X*_34_', 'Edge, high and climbing operation protection is not in place *X*_36_', and 'Improper binding of components caused by workers' illegal operation *X*_25_'. At the same time, the key risks to construction safety are concentrated in the component lifting stage, and the risks in the component lifting stage should be controlled in the construction process.

#### Prevention and control measures for high-risk factors

According to the calculation results of the risk degree of lifting operation in Table 7, the project's 'Low safety awareness causes workers to operate illegally *X*_23_', 'Operation error caused by illegal command *X*_33_', 'Workers' risky work caused by low safety awareness *X*_34_', 'Edge, high and climbing operation protection is not in place *X*_36_', and 'Improper binding of components caused by workers' illegal operation *X*_25_', and other indicators have a high degree of risk, and there are multiple accident causes of coupling. If it continues to develop, it is easy to cause lifting injury accidents^37^. Therefore, it is necessary for all construction participants to pay attention to it and take measures as shown in Table 8 below:

**Table 8:** Prevention and control strategy of high risk causes of lifting operation.

Index	Safety risk	Prevention and control strategy
*X*_23_	Low safety awareness causes workers to operate illegally	Safety education and training should be carried out before the operation of relevant operators, and a safety education assessment should be carried out. Only those who pass the assessment can work on duty
*X*_33_	Operation error caused by illegal command	Safety education and training should be carried out before the operation of relevant operators, and a safety education assessment should be carried out. Only those who pass the assessment can work on duty
*X*_34_	Workers' risky work caused by low safety awareness	Implement the ' reward and punishment mechanism ', encourage reporting violations and give corresponding rewards, and give corresponding punishments for discovered violations, so as to improve the safety and production efficiency of lifting workers
*X*_36_	Edge, high and climbing operation protection is not in place	Regularly carry out safety hazards investigations, implement ' reward mechanisms, encourage reporting safety hazards and give corresponding rewards, find safety hazards, and deal with them in time to avoid accidents caused by safety hazards
*X*_21_	Improper binding of components caused by workers' illegal operation	Safety education and training should be carried out before the operation of relevant operators, and a safety education assessment should be carried out. Only those who pass the assessment can work on duty

### Cable machine engineering risk level calculation

According to the interaction matrix data obtained in “Cable machine engineering risk level calculation” and Formulas 4 and 5, the total influence degree *C* and the total affected degree *E* are calculated. The weight of each index is calculated and sorted by Formula (7). The results are shown in Table 7. According to the formula (7), the risk value of the cable crane project is *P* = 0.4527 < 0.60, that is, the risk level of the project is grade III. This result is consistent with the actual situation of the project because it has complex geological conditions, more on-site work types and mechanical equipment, a long construction period, frequent on-site mechanical operations, and ground settlement caused by concentrated summer rainstorms. The project is in significant danger, and safety control needs to be further strengthened to ensure the safety of the lifting operation site.

## Limitations and future work

This paper may have the limitations. (1) Strong subjectivity: the risk factors identification and influencing factors of lifting operation construction in this paper are obtained through expert consultation. Different experts may have different results, which leads to certain limitations of the evaluation results; (2) Limitations of data sources: due to the complexity and uncertainty of the construction site, it is difficult to collect complete and accurate data, and the research object and example verification in this paper are based on Chinese data, data acquisition has certain limitations; (3) Lack of dynamic: The environment and conditions of the lifting construction site may change over time, but the method proposed in this paper is difficult to update and adjust in real time, and cannot reflect the dynamic changes of the construction site.

There are few studies on the influence of coupling among risk factors on lifting accidents in the text. In the future research, we will further consider the coupling effect between risk factors, and based on this, we will evaluate the importance of risk factors and study the safety status of lifting operations.

## Discussion

At the construction site, workers' violations can be monitored and reminded in real time through on-site surveillance cameras to correct workers ' unsafe behaviors in a timely manner. With the development of wearable technology, a variety of wearable devices have also been applied to the construction site, such as smart boots: By walking, smart boots can detect the risk of collisions between workers and nearby engineering vehicles equipped with sensors. Smart helmet: By sensing brain waves, smart helmets can detect 'micro-sleep', which reduces the risk of injury to workers. By wearing sensors to workers, when workers are close to dangerous areas such as borders and high places, they can detect and remind workers in time to reduce safety risks. In view of the series of unsafe behaviors and risky operations caused by the low safety awareness of workers, the most effective way is to strengthen the safety education training and assessment of workers before they go to work, and only those who pass the assessment can go to work. In addition, the effectiveness of management is an important cornerstone of construction site safety management and control. Therefore, it is necessary to strengthen the management responsibility and awareness of managers.

## Conclusion

1. Based on WBS work structure decomposition and RBS safety risk decomposition, the construction risk evaluation index system for lifting operations is constructed. Combining system theory and safety risk theory, the construction safety risk of tower building platforms is identified, and the construction risk evaluation index system of tower building platforms with 16 indexes is constructed.
2. A coupling evaluation model of lifting operation safety risk is constructed. The risk coupling theory is introduced to analyze the interaction between human factors, machine factors, ring factors, and pipe factors in the lifting operation system. An evaluation model based on the C-OWA operator and interaction matrix is constructed, and the risk level is calculated to provide a safety control basis for lifting operations.
3. Combined with the actual project case to verify the rationality of the evaluation index system and evaluation method According to the field survey data and the evaluation model, the cable crane project is calculated to be at a significant risk level, which is consistent with the actual situation. Based on the evaluation model, the causes of high-risk accidents in the project are analyzed, and suggestions for improvement are put forward so as to reduce the risk of lifting operations and ensure their safety.

## Data Availability

The datasets generated and/or analysed during the current study are not publicly available due to all the data are collected by the author, so it is not convenient to disclose but are available from the corresponding author on reasonable request.
